# Editorial: Forest soil microbiome and their interactions with the plants

**DOI:** 10.3389/fmicb.2026.1819757

**Published:** 2026-04-07

**Authors:** Irfan Ali Phulpoto, Muneer Ahmed Qazi, Jie Yang, Hesong Wang

**Affiliations:** 1 Institute of Microbiology, Shah Abdul Latif University, Khairpur, Sindh, Pakistan; 2 School of Water Resources and Environment, China University of Geosciences, Beijing, China; 3 School of Ecology and Nature Conservation, Beijing Forestry University, Beijing, China

**Keywords:** forest soil, microbiome, plant health, plant-microbe interactions, soil microbiome, soil virome

## Introduction

Forests cover approx. 38 million km^2^ of Earth's surface, and comprise billions of trees. In addition to food and timber production, the forest ecosystem also helps to sustain regional ecosystem services including reduction of carbon footprint and elemental biogeochemical cycling (Perry et al., 2008; Pan et al., 2011; Crowther et al., 2015; Li et al., 2023). Forests are extremely diverse ecosystems with a wide range of reactive interfaces mediated by microbes. These primarily include the root-soil, root-root, litter-soil, and plant-atmosphere interfaces (Kim et al., 2012; Voríšková et al., 2014; Beckers et al., 2017). All these interfaces have distinct features, such as the factors influencing microbial diversity, microbial abundance of particular microbial species, and the nutritional availability (Li et al., 2023).

Furthermore, the ecosystem activities impact the habitats of microorganisms, particularly fungi, which live or even associate with such habitats. The dynamics of forests span over a wide range of time periods, from short-term incidences during seasonal ecosystem dynamics to the long-term stand growth following disturbances including fires or insect outbreaks (Baldrian, 2017). Identifying these mechanisms require in-depth investigations of the microbiome and its functioning through targeted, integrated microbiological and ecological research conducted across different environments. The Research Topic i.e., *Forest soil microbiome and their interaction with plants*, seeks integration of interdisciplinary study that promotes a mechanistic understanding of such biological activities in these natural ecosystems under fluctuating environmental conditions. The underlying concept is that microbes in forest soil are dynamic plant symbionts contributing toward growth promotion, in nutrient processing, and improving defense and resistance to environmental stresses.

A paradigm shift may be seen in the articles collection compiled under this topic. Our understanding of forest soils is evolving from the simple chemical reactions to diverse biological interactions. One the most intriguing discoveries in this Research Topic is the crucial third player in soil ecology, i.e., soil virome in addition to bacteria and fungi, which play pivotal role as decomposers in the subtropical secondary forests soil (Chen, Yu et al.). Soil viruses are not only pathogens but they also facilitate the viral shunt (Tong et al., 2023). The flow of carbon is effectively interrupted by the virus-infected microbial hosts releasing unique signals of dissolved organic matter. Chen, Yu et al. showed how variations in bacterial metabolism during forest succession cause a significant shift in the population structure of soil viruses. This implies that viruses are active regulators of the carbon cycle influencing the ration of carbon stored in the soil to carbon emitted as CO_2_. As we seek to protect global carbon reserves, we may no longer disregard the viral component of the soil microbiome.

According to the Lladó et al. (2017), forest soils are stratified with markedly different environmental conditions and resource availability at every centimeter of depth. The impact of soil layers on microbiome diversity and their network complexity of *Pinus yunnanensis* at the Ailao Mountains subtropical forest was studied by Qiao et al.. Their findings demonstrated that the seasonal fluctuations are not as effective as soil depth, which serves as a filter for microbial community building through soil layers. The strategies for conservation should take this variation into account. Notably, Qiao's study suggests that the subsurface contains unique microbial reservoirs that operate according to various ecological norms. The majority of soil sampling is limited to the topsoil (0–10 cm). The stability of deep soil microbiomes may serve as a buffer to maintain biodiversity and recolonize topsoil to sustain ecosystem functions when surface is perturbed by anthropogenic activities and/or any changing climatic condition such as rainfall and temperature.

The stable existence of these microbial communities is being threatened by shifting precipitation patterns. Wang et al. contribution provides a warning about the forest soil's hydrological vulnerability. Through examining how *Pinus yunnanensis* microbiome react to rainfall variations, the authors found that varying moisture availability affects the complex balance between bacterial and fungal networks. Accordingly, fungi are the main decomposers of recalcitrant wood, which are very sensitive to moisture. The structure of microbial network may collapse or rearrange in response to shifts in rainfall patterns that result in prolonged droughts or more violent floods resulting in slowdown of nutrient cycling and obstructed tree development.

At the root-soil interface, i.e., rhizosphere, the most intimate interactions take place, which directly influence the indigenous microbiome. More importantly, the plants are active architects not passive consumers of soil nutrients. According to Xie et al., the degraded soil health frequently results from dysbiosis or an imbalance in the microbial diversity of mango orchards infected with soilborne diseases. However, the plants strive to protect themselves by secreting root exudates to stimulate beneficial microbial community and inhibit pathogens (Rolfe et al., 2019). Similarly, Chen, Cao et al. found an explicit association between certain soil microbes and the nutrient value of bamboo shoots in their study on *Dendrocalamus brandisii*. This expands the research to quality measures rather than merely growth indicators. It implies that the forest microbiome also regulates the texture, flavor, and nutritional content of plants and associated products. Ultimately, a healthy forest ecosystem may be determined by the interplay at plant-microbe interface.

Pfeufer et al. concentrated on *Oomycetes* (water molds), which are responsible for some of the most catastrophic plant diseases of the past such as the Irish Potato famine. Based on their investigation, *Phytophthora* and *Phytopythium* species are abundant in both wild and agricultural regions in Vietnam. For biosecurity, it is crucial to comprehend the global dissemination of these cryptic pathogens to forest with no prior exposure to these pathogens and therefore lack the co-evolved microbial defenses when global trade moves soil and plant material across borders (Lovett et al., 2016).

These findings not only improve our basic understanding of the diversity and functions of the forest soil microbiome but also provide the groundwork for integrating microbial ecology into practical approaches for the forest management and conservation. For instance, understanding how soil microbial networks get impacted by rainfall patterns and climate change could potentially guide responsive forest management in the context of changing climate conditions. Comprehending the feedbacks between plants and forest soil also offers possibilities to utilize favorable microbial interactions for improving plant health, disease resistance, and the resilience of ecosystem. As topic editors, we are pleased by the depth of the research contribution in this Research Topic. These findings emphasize the significance of ongoing integrated research that involves field observations, functional omics, high resolution sequencing, and experimental manipulations to unravel the intricate relationship between forest plants and their related soil microbiome. We recommend readers to investigate these contributions in depth and engage with the emerging issues they raise. Ecosystem stability, plant health, and global biogeochemical cycling are all shaped by forest soil microbiome, which is more than just a background for ecological interactions. In order to address the future scientific and environmental concerns, it will be essential to continue investigating at these systems.
